# Influence of maternal socioeconomic deprivation and living environment on newborn bloodspot 25-hydroxyvitamin D levels

**DOI:** 10.3389/fendo.2022.978580

**Published:** 2022-11-03

**Authors:** Wolfgang Högler, Katharina Tischlinger, William D. Fraser, Jonathan C. Y. Tang, Suma Uday

**Affiliations:** ^1^ Department of Paediatrics and Adolescent Medicine, Johannes Kepler University Linz, Linz, Austria; ^2^ Institute of Metabolism and Systems Research, University of Birmingham, Edgbaston, Birmingham, United Kingdom; ^3^ Department of Medicine, Norwich Medical School, University of East Anglia, Norwich Research Park, Norwich, United Kingdom; ^4^ Departments of Diabetes and Endocrinology and Clinical Biochemistry, Norfolk and Norwich University Hospital, Norwich, United Kingdom; ^5^ Clinical Biochemistry, Departments of Laboratory Medicine, Norfolk and Norwich University Hospital NHS Foundation Trust, Norwich, United Kingdom; ^6^ Department of Endocrinology and Diabetes, Birmingham Women’s and Children’s Hospital, Birmingham, United Kingdom

**Keywords:** income, vitamin D (25-hydroxyvitamin D), employment, housing, micronutrients, neonate - age

## Abstract

**Objectives:**

Vitamin D deficiency in neonates can have life-threatening consequences, hence the knowledge of risk factors is essential. This study aimed to explore the effect of maternal socioeconomic status (SES) on newborn 25-hydroxyvitamin D (25OHD) concentrations.

**Design:**

Over two 1-week periods (winter and summer of 2019), 3000 newborn heel prick dried blood spots (DBS) and additional data of newborns, from a regional newborn screening laboratory (52° N) in the West Midlands, UK, were gathered. Post code was replaced with lower layer super output area (LSOA). Index of Multiple Deprivation (IMD) quintiles for the corresponding LSOA was used to assess SES [quintile one (Q1): most deprived 20%, quintile five (Q5): least deprived 20%]. Each of the seven domains of deprivation were examined (income, employment, education, health, barriers to housing and services, crime and living environment). 25OHD was measured on 6mm sub-punch from DBS using quantitative liquid chromatography tandem mass spectrometry and equivalent plasma values were derived.

**Results:**

In total 2999 (1500 summer-born, 1499 winter-born) newborn DBS (1580 males) were analysed. Summer-born newborns had significantly higher 25OHD (IQR) concentrations [49.2 (34.3; 64.8) nmol/l] than winter-born newborns [29.1 (19.8; 40.6) nmol/l, p<0.001].25OHD levels varied significantly between the different IMD quintiles in the whole (p<0.001) and summer-born cohort (p<0.001), but not in the winter-born cohort (p=0.26), whereby Q1 had the lowest 25OHD concentrations. Among the domains of deprivation, living environment had a significant influence on 25OHD levels (β=0.07, p=0.002). In this subdomain, 25OHD levels varied significantly between quintiles in the whole (p<0.001) and summer-born cohort (mean 25OHD Q1 46.45 nmol/l, Q5 54.54 nmol/l; p<0.001) but not in the winter-born cohort (mean 25OHD Q1 31.57 nmol/l, Q5 31.72 nmol/l; p=0.16). In a regression model, living environment was still significant (p=0.018), albeit less than season of birth and ethnicity.

**Conclusion:**

Among the seven domains of deprivation, maternal living environment had the greatest effect on newborn 25OHD levels. Whilst improved living environment positively influenced vitamin D status in the summer-born babies, winter-born had low 25OHD levels irrespective of the environment. Strategies such as enhanced supplementation and food fortification with vitamin D should be considered to overcome the non-modifiable main risk factors for vitamin D deficiency.

## Introduction

Akin to many other micronutrients, the newborn’s vitamin D status depends mainly on maternal supplies. Adequate maternal vitamin D reserve confers protection of her offspring from complications of vitamin D deficiency and provides adequate mineral substrate for bone growth, strength and wellbeing (1–4). Infants are a known risk group for vitamin D deficiency for several reasons, including deprivation of direct sunlight for cutaneous production (5), rapid growth and the lack of adequate vitamin D in breast milk (6). Vitamin D deficiency can lead to serious hypocalcaemic and hypophosphataemic complications. Hypocalcaemic complications in infancy include seizures, tetany, dilated cardiomyopathy and cardiac failure (7–9). Hypophosphataemic complications typically manifest after the first 6 months of life and can lead to poor growth, delayed motor milestones and nutritional rickets (10).

Ethnicity, living at high latitude, season, reduced sun exposure or chronic diseases are just a few well studied risk factors for vitamin D deficiency (11). It has been well recognised that socioeconomic status (SES), affects the prevalence of obesity and care of children with type 1 diabetes (12, 13), similarly it is thought to affect vitamin D status. Heterogeneity of SES definitions and confounding factors such as diet and lifestyle are likely to influence the association. To assess deprivation some studies use an overall SES index or index of multiple deprivation (IMD) (14–16) and others differentiate between its components such as income, employment, housing or education (17, 18).

There is evidence that socioeconomic deprivation, which is assessed in various ways in the literature such as SES index or individual income data, is associated with lower 25OHD levels (19–21). Data from pregnant women in the US showed an association of maternal 25OHD with SES, but after multivariable analysis including ethnicity, vitamin D supplement use and physical activity, the association with SES was no longer evident (22).

Whether newborn 25OHD level is influenced by maternal SES is not entirely clear. Some studies observed an association between neonatal vitamin D status and different maternal SES components such as health insurance status (18) and housing (23). However, other studies showed no association of 25OHD levels with maternal SES as assessed by education and annual income, following single variant analysis (24), or after multivariable analysis including ethnicity, season of birth and prenatal vitamin D intake (17).

Here, we set out to assess the influence of maternal SES on newborn vitamin D status in a large cohort of summer- and winter-born babies from diverse ethnic backgrounds.

## Aims

1. Ascertain the variation in newborn 25OHD levels based on maternal deprivation quintiles.
2. Evaluate the influence of each of the seven domains of maternal deprivation quintiles (income, employment, education, health, crime, barriers to housing and services and living environment) on newborn 25OHD levels.
3. Evaluate the seasonal variation in influence of living environment quintiles on newborn 25OHD levels.

## Research design and methods

### Study design:

3000 dried blood spot (DBS) cards received at Birmingham Women’s and Children’s Hospital’s regional newborn screening laboratory, covering the West Midlands region of England (52° N), were collected after obtaining ethical and regulatory approvals. After anonymisation of the data collected on newborn blood spot (NBS) cards, quantitative liquid chromatography tandem mass spectrometry was used to measure 25OHD concentration on DBS and evaluate plasma equivalent values. Results on the ethnic and seasonal variations in 25OHD of the cohort have been published (25). Here, we present the results of additional analysis focusing on socioeconomic data.

### Study population:

To identify a panel of metabolic diseases, the national NBS programme screens every newborn baby in England by collecting a DBS sample around day 5 of life. After all routine NBS testing was done these samples were gathered in two one-week periods, one in winter (February 2019) and one in summer (August 2019). Samples with high risk of infection, incomplete data or insufficienct sample, second tests and newborns older than 21 days were excluded.

### DBS samples:

An appropriately trained health care professional obtained the sample by a heel prick and transferred it to a standard Whatman 903 filter paper. The samples were continuously kept at room temperature and away from direct sunlight during daily transportation from the maternity service to the screening laboratory. All samples arrived in the screening laboratory within 3 days and 90% on the same day. Within a week after arrival at the screening laboratory the samples were stored at -20°C and analysis undertaken at recruitment completion (October 2019-December 2019) (25).

### Source data

Information about the newborn (birth weight, gestational age) and the mother (maternal age, ethnic group code) was obtained and anonymised. Ethnic group codes were assigned (**Box 1**) according to the UK Office of National Statistics (26).

### Lower Layer Super Output Area (LSOA)

Postcode was replaced with LSOA, a geographic area used for small area statistics. Every LSOA contains a mean population of 1500, yielding to a total of 32,844 LSOA’s in England and every LSOA has a deprivation index rank assigned (27).

### Deprivation indices

The UK government uses 39 separate indicators to measure indices of deprivation. The Index of Multiple Deprivation (IMD) is part of the Indices of Deprivation and is the most widely used of these indices. It combines information from the seven domains (which measure different types or dimensions of deprivation) to produce an overall relative measure of deprivation. The seven main domains with different impact include income (22.5%), employment (22.5%), education (13.5%), health (13.5%), crime (9.3%), barriers to housing and services (9.3%) and living environment (9.3%) (27).

Every LSOA can be ranked according to their IMD (1 to 32,844) and each subdomain from the most deprived to the least deprived or it can be divided in five quintiles. Each quintile accounts for 20% of the total LSOA with quintile (Q)1 indicating the most deprived, Q2 the second most deprived, Q3 the third least deprived, Q4 the second least deprived and Q5 the least deprived (28).

Box 1 **Ethnic group codes according to the UK Office of National Statistics.**

**Table:** 

Group code	Ethnicity
1	White British
2	Any other White (White Irish and any other White)
3	Asian (Indian, Pakistani, Bangladeshi, Chinese and any other Asian background)
4	Black (African, Caribbean and any other Black background)
5	Mixed White and Black (White and Black African and White and Black Caribbean)
6	Mixed White and Asian
7	Any other mixed (any other mixed background and any other ethnicity)

### 25 OHD analysis and plasma equivalent values

The Bioanalytical facility of the University of East Anglia, Norwich, UK, performed the analysis of 25OHD3 and 25OHD2 levels of the DBS cards by using quantitative liquid chromatography tandem mass spectrometry followed by extraction methods as previously described (29, 30).

Using a DBS card puncher (Analytical Sales & Services, NJ, USA) an individual 6mm part of each DBS card was collected and then further processed by extracting samples using 300µL of 50:50 (v/v) isopropanol to water solution containing carbon-13 labelled 25OHD3-^13^C_5_ internal standard. This was kept in an ultrasonic water bath at 35°C for 30 minutes. A supported Liquid Extraction plate (Biotage, Uppsala, Sweden) was used as medium for further purification of the extracts followed by elution with 1.5mL of heptane. Subsequent processing included drying, derivatization and detection using Micromass Quattro Ultima Pt tandem mass spectrometer (Waters Corp., Milford, MA, USA) as detailed previously (25).

Quality controls prepared in vitamin D and its hydroxylated metabolite-free packed red cells as well as a matrix-matched calibration standard was used for analysis of the samples.

As reported previously (25) across the concentration range of the assay there was an inter/intra-assay coefficient of variation between 3.9-9.4% and linearity from lower limit of quantification of 1 nmol/l to 150 nmol/l.

Equivalent total plasma 25OHD was derived using the following calculation (25):


Plasma equivalent total 25OHD nmol/L=[DBS (25OHD3 +25OHD2)−1.2607]/(1–0.60).

As 25OHD is mainly present in the extracellular fluid compartment adjustment for haematocrit was included. Owing to the higher Hct in neonates (31) and capillary blood samples (32) a factor of 0.60 was used.

### Ethics and consent

The UK Health Research Authority, the East Midlands – Leicester South Research Ethics Committee (REC Reference 19/EM/0019) and the Antenatal and Newborn Research Advisory Committee of Public Health England, UK approved the study. In accordance with the code of practice for retention, storage and use of residual NBS, parental consent was not needed (33).

### Statistical analysis

For descriptive statistics, median with interquartile ranges (IQR) or mean with standard deviation (SD) was used for continuous variables and frequencies with percentages were used for categorical variables. 25OHD levels were used as both continuous and categorical variable. Vitamin D deficiency was defined as 25OHD concentrations<30 nmol/L (12 µg/l), insufficiency as 30-50 nmol/L (12-20 µg/l) and sufficiency as >50 nmol/L (>20 µg/l) in line with the Institute Of Medicine (34) and Global Consensus Recommendations (35). Deprivation quintiles (1–5) were used as categorical variables.

As 25OHD levels were not normally distributed, non-parametric tests were used. After root transformation a normal 25OHD distribution was attained. Because results on parametric tests were very similar, these are not reported here.

Kruskal-Wallis test was used to compare 25OHD concentrations between deprivation quintiles with further pair-wise analysis to ascertain differences between groups. Stepwise linear regression was used to assess the effect of each of the seven components of deprivation on total 25OHD levels. Additional independent variables (season of birth, ethnicity, gestational age, maternal age) which were significant on bivariate analysis were also entered in the regression analysis to ascertain the effect of deprivation on total 25OHD in the full model. SPSS statistical software v28.0 (IBM, Armonk, NY) was used for all analyses.

## Results

### General demographics

2999 samples (1499 winter-born, 1500 summer-born) were included in 25OHD analysis of whom 1580 were male. The age of the newborn at sample collection ranged between 5 and 19 days, but 99% of the samples were collected within the first 7 days of life.

More than half of the newborns were white British (59.1%), had a mean birth weight of 3306 (± 565) grams and were born at term at 38.3 (± 1.8) weeks of gestation. The descriptive characteristics of the study cohort, as previously published (25), are shown in **Table 1**.

**Table 1:** Baseline characteristics of the study cohort with subanalysis of winter-borns and summer-borns.

	Whole cohort	Winter-born	Summer-born	p-value
n	2999	1499 (one missing)	1500	
Number of males (% of whole cohort)	1580 (52.7%)	771 (51.4%)	809 (53.9%)	0.16
Birth weight in g (± SD)	3306 (565)	3313 (563)	3299 (566)	0.49
Gestational age in weeks (± SD)	38.8 (1.8)	38.8 (1.8)	38.8 (1.7)	0.57
Maternal age in years (± SD)	30.4 (5.5)	30.4 (5.6)	30.5 (5.4)	0.61
Ethnic groups n (%)				0.64
White British	1774 (59.1%)	877 (58.5%)	897 (59.8%)	
Any other White	264 (8.8%)	134 (8.9%)	130 (8.7%)	
Asian	494 (16.5%)	249 (16.6%)	245 (16.4%)	
Black	173 (5.8%)	90 (6.0%)	83 (5.5%)	
Mixed White and Black	94 (3.1%)	53 (3.5%)	41 (2.7%)	
Mixed White and Asian	45 (1.5%)	21 (1.4%)	24 (1.6%)	
Any other mixed	156 (5.2%)	76 (5.1%)	80 (5.3%)	

### Season, ethnicity and 25OHD levels

These results have been published in detail (25) but are briefly summarised below. The overall median (IQR) 25OHD level was 37.8 (24.8; 54.8) nmol/l. 25OHD deficiency was noted in 35.7% (n=1070), insufficiency in 33.7% (n=1010) and sufficiency in 30.6% (n=919) of the DBS cards. The summer-born cohort had significantly higher 25OHD concentration [49.2 (34.3; 64.8) nmol/l] compared to the winter-born cohort [29.1 (19.8; 40.6) nmol/l, p<0.001]. The median (IQR) 25OHD level was significantly higher in newborns of White British descent [41.6 (27.6; 59.3) nmol/L], compared to all other groups, with the lowest 25 OHD levels in the newborns of Black ethnic background [30.3 (19.4; 43.2) nmol/L, p<0.001].

### 25OHD and deprivation

**Figure 1:**
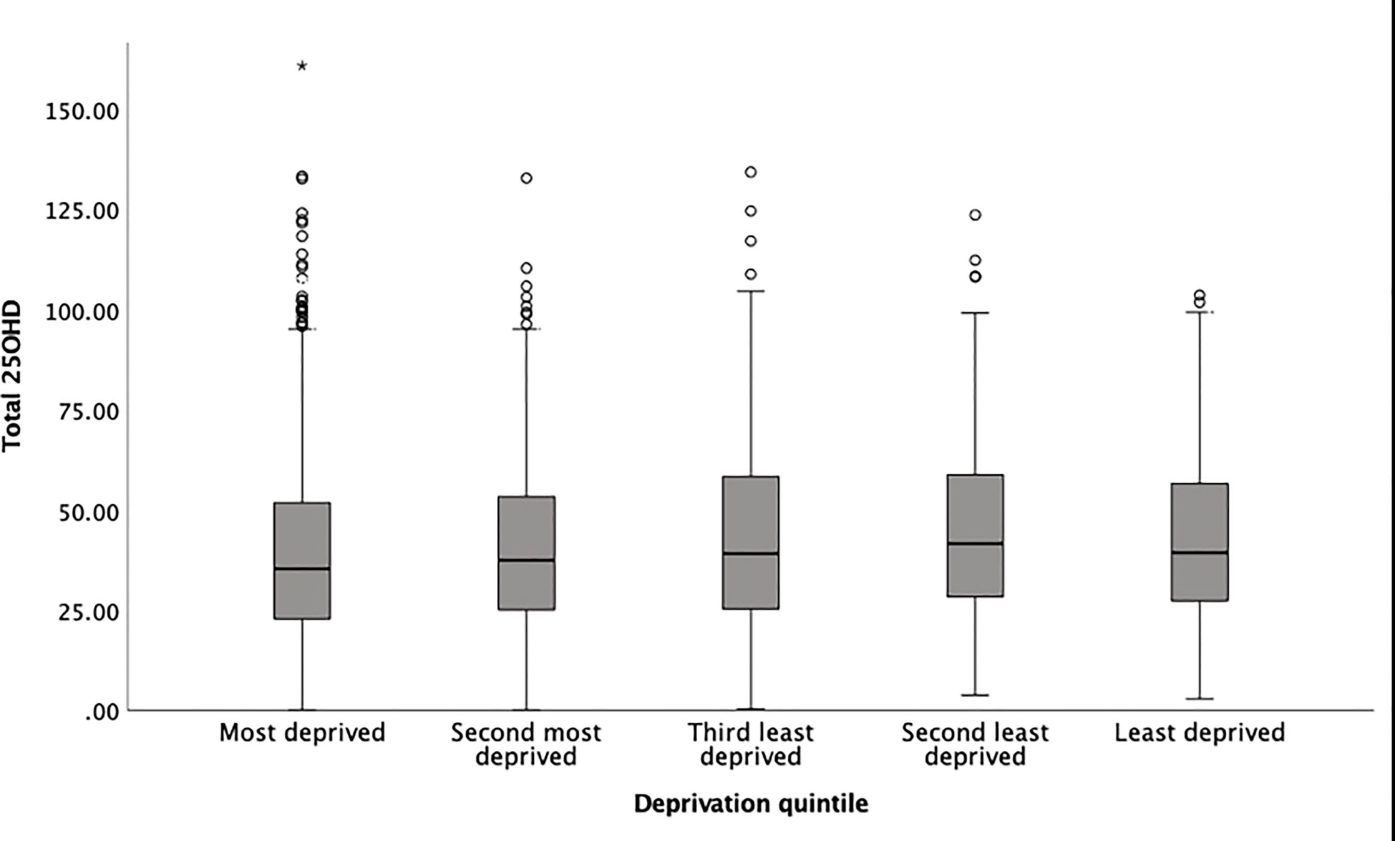
Box plot of newborn 25OHD concentrations from DBS cards and quintiles of IMD (total study cohort). * is an outlier.

**Figure 2:**
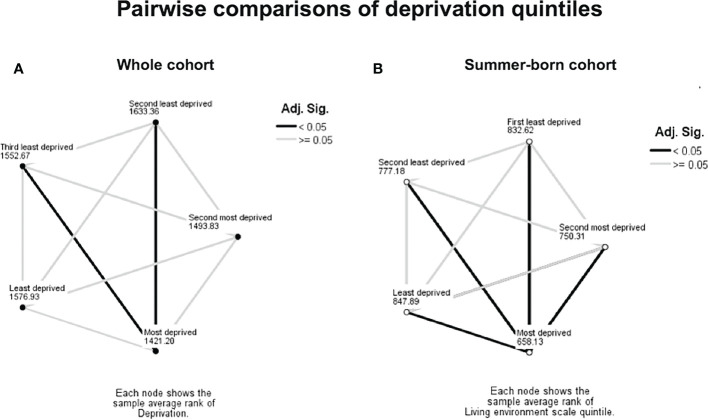
Pairwise comparison between socioeconomic groups, whole **(A)** and summer-born cohort **(B)**. Numbers represent IMD average ranks, dark lines represent significant difference (p<0.05).

The variation in 25OHD levels between the IMD quintiles was statistically significant in the whole cohort (p<0.001, **Figure 1** and **Table 2**) and in the summer-born cohort (p<0.001) but not in the winter-born cohort (p=0.26). A pairwise comparison demonstrated significant differences (p<0.05) in 25OHD levels between specific IMD quintiles in the whole (**Figure 2A**) and summer-born (**Figure 2B**) cohort but not in the winter-born cohort.

**Table 2:** Newborn 25OHD concentrations grouped in IMD quintiles.

	Mean (SD) 25OHD nmol/L	Median (IQR) 25OHD nmol/L
Most deprived (n=1221)	39.4 (22.3)	35.3 (22.8; 52.0)
Second most deprived (n=590)	40.7 (21.0)	37.5 (25.0; 53.3)
Third least deprived (n=526)	42.2 (22.4)	39.1 (25.3; 58.4)
Second least deprived (n=376)	44.3 (22.1)	41.6 (28.3; 58.8)
Least deprived (n=286)	42.5 (21.0)	39.3 (27.2; 56.6)

**Figure 3:**
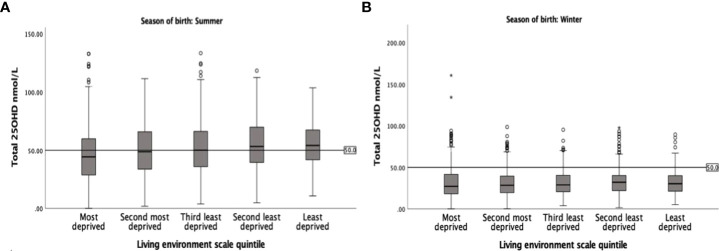
Box plot of 25OHD levels in the summer-born **(A)** and the winter-born **(B)** cohort of the different living environment quintiles. Note the variation in 25OHD levels by the different living environment quintiles in the summer-born and the persistently low 25OHD levels in the winter-born cohort.

**Figure 4:**
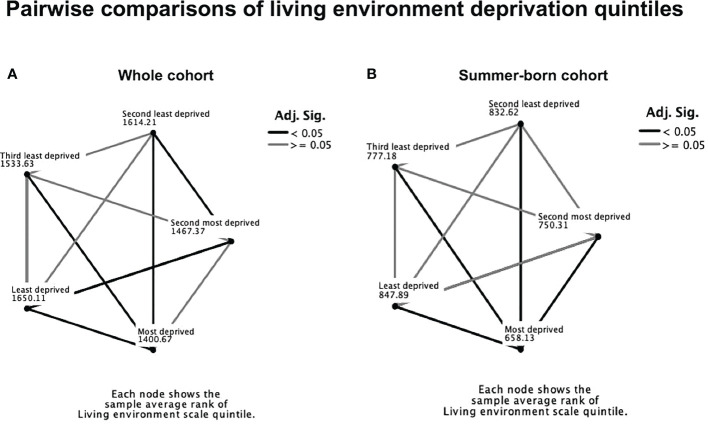
Pairwise comparison of living environment quintiles in the whole cohort **(A)** and in the summer-born cohort **(B)**. Numbers represent IMD average ranks, dark lines represent significant differences (p<0.05).

Living environment was identified as the most significant domain of deprivation influencing 25OHD levels (β=0.07; p=0.002) among the seven independent domains of deprivation (**Table 3**). 25OHD levels varied significantly between different living environment quintiles in the whole (p<0.001) and summer-born cohort (p<0.001, **Figure 3A**) but not in the winter-born cohort (p=0.16, **Figure 3B**). A pairwise comparison demonstrated significant (p<0.05) differences in 25OHD levels between the different living environment quintiles in the whole (**Figure 4A**) and summer-born (**Figure 4B**) cohort but not in the winter-born cohort. In a multivariable regression model (**Table 4**), season of birth, ethnicity, maternal age, gestation of birth and living environment deprivation quintile were statistically significant factors affecting 25OHD levels. Season of birth, ethnicity and gestational age explained the greatest part (23.1%) of the variation in 25OHD level, whilst maternal age and living environment quintile each explained only another 0.1% of the variation

**Table 3:** Different domains of IMD and their effect on 25OHD in the whole cohort.

	Standardized CoefficientsBeta	Sig.
(Constant)		<0.001
Deprivation quintile	-0.073	0.301
Income scale quintile	0.131	0.051
Employment scale quintile	-0.074	0.215
Education scale quintile	0.000	0.990
Health disability scale quintile	0.046	0.253
Housing scale quintile	0.035	0.106
Living environment scale quintile	0.069	0.002

**Table 4:** Multivariable analysis (dependent variable 25OHD) with different models indicating living environment as a significant factor affecting 25OHD levels.

	Standardized Coefficients Beta	Sig.p
Season of birth	0.435	<0.001
Ethnic groups	-0.162	<0.001
Gestational age	-0.118	<0.001
Maternal age	0.043	0.008
Living environment scale quintile	0.039	0.018

### Ethncitiy and deprivation

**Figure 5:**
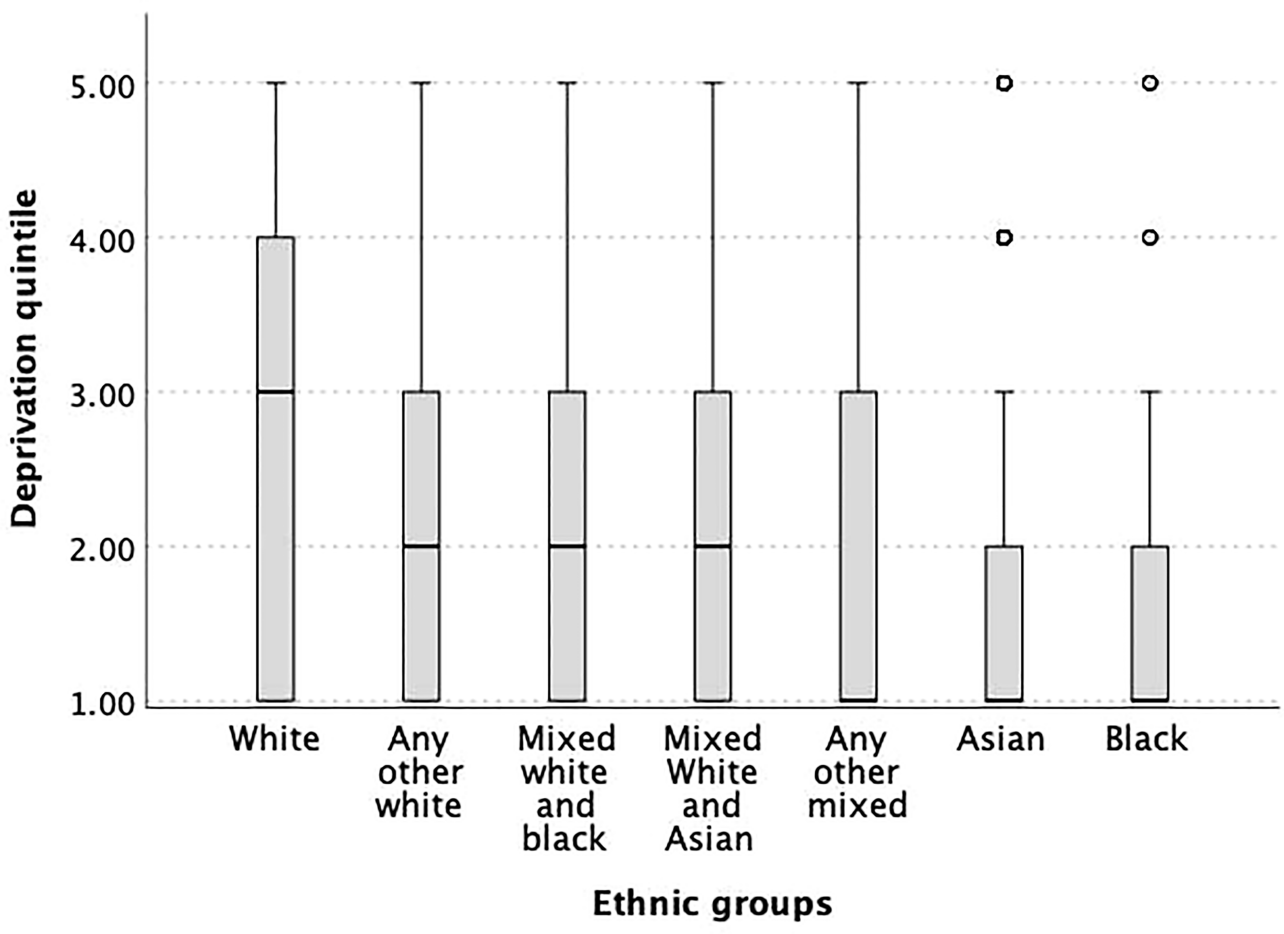
Box plot of the different ethnicities and associated deprivation quintiles indicating strong differences of SES in the different ethnic groups. All ethnicities, except for the mixed White and Asian, had significantly lower newborn 25OHD levels than White ethnicity (p<0.05).

The median deprivation quintile in all ethnic groups, except mixed White and Asian, was significantly lower when compared to the White ethnic group (**Figure 5**).

## Discussion

In this multi-ethnic large newborn cohort, only 30% of the newborns had sufficient 25-hydroxyvitamin D levels, with highest levels in newborns of White ethnic background and those born in summer. IMD, which was lowest in babies of Black, Asian and any other mixed ethnic origin, was significantly associated with lower 25OHD levels. We demonstrate that better living environment positively influences 25OHD levels in summer-born babies but the winter-born had consistently low 25OHD levels confirming the substantial effect of season on vitamin D status. Despite the strong effect of season and ethnicity on 25OHD levels, living environment still remained significant in a multivariable regression.

We found that the IMD influences the vitamin D status in the overall and summer-born cohort, but not in the winter-born cohort. Of the seven different domains of deprivation, living environment was the strongest predictor of a newborn’s 25OHD level primarily affecting the summer-born cohort, indicating that indoor living and sun exposure play a substantial role during pregnancy. The observation that living environment is a strong predictor of an individual’s vitamin D status has been reported in previous studies (36, 37). Living environment has been shown to be a statistically significant predictor of vitamin D status even after adjusting for ethnicity and season (37), an association which was reproduced in our study. Assessment of living environment includes indoor aspects, such as housing in poor conditions or failing to meet decent homes standard, and outdoor aspects like air quality (38). Attractive neighbourhood environment is associated with increased leisure time and physical activity (39). Reduced physical activity or unattractive leisure time facilities are associated with reduced time spent outdoors and thus reduced UVB exposure (40). The effect of living environment, in our cohort, was reduced in the winter months since the necessary UVB spectrum for cutaneous vitamin D production is missing from UK sunlight between October and March. Vitamin D winter therefore affects newborns of all living environment quintiles (41). However, we do not have the data to confirm different UVB exposure in the cohorts from different deprivation quintiles.

The relationship of UVB exposure and SES is quite difficult to assess. Several factors have to be addressed when evaluating outdoor activity and therefore UVB exposure, for example occupation, residency, leisure time activities or transport physical activity like cycling or walking to work. Greater leisure time outdoor physical activity has been associated with higher SES in rural, suburban and urban residents (42, 43). However, occupational physical activity and thus time spent outdoors may affect all SES groups (44).

Maternal factors associated with neonatal vitamin D deficiency other than SES have been previously investigated in studies utilising DBS and have demonstrated a correlation with ethnicity, sunlight exposure prior to delivery (45), ambient temperatures or sunshine hours (46).

In the UK, the relation of SES and ethnicity is evidenced by the fact that people of white ethnic origin are more likely to live in the least deprived areas than people from other ethnic groups (47). Previous studies from Birmingham reported a significant positive correlation between the percentage of non-white population and socioeconomic deprivation, but no correlation between median 25OHD concentration and IMD after multivariate analysis (16). This finding supports the notion that ethnicity is a stronger predictor of vitamin D status compared to IMD. In our cohort living environment continued to be statistically significant in regression model including season of birth and ethnicity, but to a lesser extent than the two latter ones. We examined the link between ethnicity and IMD and found that people of white ethnic background had significantly higher IMD when compared to all other ethnic groups except the mixed White and Asian group.

Our study’s strengths include the large sample size and no missing data for the studied parameters. Limitations of this study are the missing information on supplement use and data on sunlight exposure. Maternal vitamin D supplementation (48) and sunlight exposure (49, 50) are associated with higher 25OHD levels in the newborn (18). The association between low SES and vitamin D supplement use is not well studied, indeed data are sparse. A survey in Ireland demonstrated no difference in vitamin D supplementation adherence between women of higher education and higher family income to those of poor education or lower family income (51). However general data on supplement use and SES indicates, that higher SES is associated with higher adherence to supplement use (52–54). Another limitation is that the IMD was used as a proxy for postcode which is less accurate, but acceptable for larger scale studies like ours. The IMD is not an absolute but rather a relative measurement, so care has to be taken when comparing the data over time or with other areas. Measurements of 25OHD in DBS provide a reliable and accurate reflection of serum 25OHD, when appropriately adjusted and storage time does not affect 25OHD concentrations (55, 56).

The public health challenge is to overcome social disparities and provide every newborn/infant as well as their mothers with sufficient vitamin D. One way is to implement vitamin D supplementation in all pregnant women and infants. Adequate 25OHD levels in pregnant women are a key factor for neonatal vitamin D stores and supplementation of pregnant women can improve the newborn’s vitamin D level effectively (48, 57–59). Most European countries have policies regarding vitamin D supplementation of infants and pregnant women, but these differ substantially (60).

Another way of addressing widespread vitamin D deficiency is food fortification, which is an elegant, cheap universal way and independent of issues related to supplementation (logistics, adherence, costs) (61, 62). Data from Finland are a prime example of how food fortification could improve 25OHD levels (40, 63). The Finish National Nutrition Council introduced a voluntary food fortification programme and achieved a substantial rise in 25OHD levels over time by fortifying milk and fat spreads and by an increase in supplement users (11% in 2000 to 41% in 2011). This intervention reduced the prevalence of vitamin D insufficiency or deficiency independent of season by increasing 25OHD levels by approximately 20nmol/L (63). USA and Canada also, but to a lesser extent, fortify food (mainly milk) with vitamin D and have achieved an improvement in population vitamin D status (64, 65). A similar approach in vitamin D fortification should follow, especially in high latitude countries. Models for successful food fortification programmes are available and an easy and cost effective way to prevent complications associated with vitamin D deficiency (66).

## Conclusions

The extent of neonatal vitamin D deficiency in the UK is worrying, since vitamin D deficiency is easy to avoid when complications are serious and potentially fatal. Knowledge about risk factors is key for prevention. SES, particularly living environment, is a risk factor, but to a lesser extent than season and ethnicity. Seasonal variation in UVB radiation and therefore endogenous 25OHD production seems to minimize the effect of living environment on 25OHD levels, since winter-born newborns had persistently low 25OHD concentrations regardless of their living environment. A holistic intervention to reach every newborn, irrespective of their SES, ethnicity or seasonal variation in UVB availability, is needed and food fortification would be an easy approach to overcome these issues. Concurrently, supplementation for infants and other high risk groups such as pregnant women should be monitored and regulated by the health care system, since these high risk groups would not benefit from fortification alone.

## Data availability statement

The original contributions presented in the study are included in the article/supplementary material. Further inquiries can be directed to the corresponding author.

## Ethics statement

The studies involving human participants were reviewed and approved by UK Health Research Authority, East Midlands – Leicester South Research Ethics Committee, UK, Antenatal and Newborn Research Advisory Committee of Public Health England, UK. Written informed consent from the participants’ legal guardian/next of kin was not required to participate in this study in accordance with the national legislation and the institutional requirements.

## Author contributions

**SU:** Funding acquisition, methodology, supervision, data curation, formal analysis, visualization and original draft writing. **WF and JT:** Investigation, sample analysis, data curation, resources and review and editing of manuscript. **WH:** Conceptualization, funding acquisition, methodology, supervision, visualization and intellectual editing of manuscript. **KT:** original draft writing, manuscript preparation and final revisions. All authors contributed to the article and approved the submitted version.

## Funding

SU was funded by a Global challenges research scholarship by the University of Birmingham, Birmingham, UK. SU and WH received additional funding for consumables from Internis pharmaceuticals limited, c/o Thornton and Ross ltd. The funders have not had any input into the design, conduct and reporting of the study. Submission was funded by Kepler University Hospital GmbH, Linz, Austria.

## Acknowledgments

We would like to thank Sunia Naseem and Jamie Large, medical students for help with sample collection. We are grateful to Russell Denmeade for supervision of sample collection and data anonymisation. We gratefully acknowledge the support of Mary Anne Preece, the director of newborn screening and Philippa Goddard, the newborn screening consultant clinical scientist.

## Conflict of interest

SU, WH and WF have previously received speaker’s fee from Internis pharmaceuticals limited, c/o Thornton and Ross ltd.

The remaining authors declare that the research was conducted in the absence of any commercial or financial relationships that could be constructed as a potential conflict of interest.

## Publisher’s note

All claims expressed in this article are solely those of the authors and do not necessarily represent those of their affiliated organizations, or those of the publisher, the editors and the reviewers. Any product that may be evaluated in this article, or claim that may be made by its manufacturer, is not guaranteed or endorsed by the publisher.
